# Dataset of the effect of the number of injector holes on the heat transfer coefficient and the pressure in the combustion chamber of a hydrogen-diesel engine

**DOI:** 10.1016/j.dib.2024.110597

**Published:** 2024-06-13

**Authors:** Javad Zareei, Jose Ricardo Nuñez Alvarez

**Affiliations:** a Department of Biosystem Engineering, Ferdowsi University of Mashhad; b Energy Department, Universidad de la Costa, Barranquilla, Colombia

**Keywords:** Heat transfer coefficient, AVL Fire, Injector nozzle, Computational fluid dynamics, Pressure

## Abstract

There are several methods for simulating internal combustion engines. The computational fluid dynamics method is the best way to simulate these engines because it can simulate the combustion process, which is a microscopic process. In this study, the simulation of the combustion process in a closed cycle in a diesel engine with a mixture of diesel and hydrogen is done by AVL Fire software. In order to simulate the combustion in the Species and chemical transmission section, a chemkin mechanism is coupled with AVL Fire software. In this study, the effect of 10 % hydrogen fuel and 90 % diesel fuel as well as the effect of nozzle holes (1, 3 and 6 holes) on the engine performance were directly investigated. In order to validate the results of the pressure simulation and the temperature inside the cylinder in the diesel fuel combustion mode, at 2800 rpm and 100 % load, the data were compared with the experimental data. The research also included verification of the heat transfer coefficient (HTC) results with theoretical data obtained by Woschni and Hohenberg. To accurately simulate the combustion process, the simulation data was validated by comparing the pressure and temperature inside the cylinder at a specific operating condition with experimental data. The results indicate that the maximum heat transfer coefficient is achieved at the angle of maximum pressure, with the exhaust valve having the highest coefficient. The addition of hydrogen to diesel fuel results in a 1.72 % increase in the heat transfer coefficient due to increased collisions. In addition, the introduction of hydrogen fuel increases cylinder pressure and engine power, while increasing the number of fuel nozzle holes decreases the coefficient and pressure, which affects fuel penetration and evaporation rate.

Specifications Table

**Table:** 

Subject	Automotive Engineering and Renewable Energy
Specific subject area	Using computational fluid dynamics to study renewable energy and fuel injection methods in the combustion chamber.
Data format	Raw, Analysed, Filtered
Type of data	Table, Image, Chart, Graph, Figure
Data collection	For collecting data, The process of mesh generation for fluid dynamics calculations involves structuring the geometry, which involves dividing the combustion chamber into different parts, such as the bed, valve seat surface, and exhaust and intake valves. The mesh for each component is generated using commercial software, such as AVL FIRE software and Chamber modeler, and then these meshes are joined together in the model assembler section to complete the process. The total number of mesh cells for the geometry was approximately 1220,000. The number of cells in the BDC (bottom dead center) was 750,000, while the number in the TDC was approximately 500,000. The total number of intake and exhaust outlet cells was 120,000, and the intake and exhaust valves and their mobility are included in the exhaust outlet passage, which had 350,000 cells. For collecting experimental data, a magnetic dynamometer is used to control the speed and load applied to the engine. The dynamometer applies an adjustable braking force against the rotation of the engine output shaft. A 500 kg load cell sensor is used to measure the torque, which is the rotational force generated by the engine. Also, an AVL Indi modul 621 piezoelectric pressure sensor was used to measure the pressure inside the chamber.
Data source location	All data were collected during 2023 by AVL Fire software and experimental data were collected at Ferdowsi University of Mashhad and de la Costa University. First location was Department of Biosystem Engineering, Ferdowsi University of Mashhad. Mashhad, Iran. Second location was Energy Department, Universidad de la Costa, Barranquilla, Colombia.
Data accessibility	Repository name: Mendeley Data Data identification number: 10.17632/6m4v5zc7v3.1 Direct URL to data: https://data.mendeley.com/datasets/6m4v5zc7v3/1
Related research article	Javad Zareei and Jose R.Nuñez Alvarez, Analysis of the effect of the number of injector nozzles on the pressure and heat transfer coefficient in a hydrogen-diesel mixture diesel engine, International journal of hydrogen energy, 2023

## 1 Value of the Data

The data obtained from this study on the simulation of the combustion process in a diesel engine with a diesel-hydrogen mixture using AVL Fire software is valuable for several reasons:

• This dataset set is valuable due to simulation provides a detailed understanding of the combustion process in a closed cycle of a diesel engine, especially focusing on the microscopic aspects. This insight is critical to improving the efficiency and performance of internal combustion engines.
• This dataset can be used as a research reference because this information is valuable in assessing the feasibility and potential benefits of alternative fuel blends in internal combustion engines, contributing to ongoing efforts to reduce dependence on conventional fuels.
• This study response influence of nozzle orifice on performance, the effect of nozzle holes (1, 3, and 6 holes) on engine performance provides valuable data for optimizing fuel injection strategies. Understanding how different nozzle configurations affect combustion can lead to more efficient and cleaner engine designs.
• Dataset can be used for the development and validation of Experimental and Simulation Data, Comparing simulation results to experimental data, particularly with respect to cylinder pressure and temperature, increases the reliability of the simulation. This validation is critical to ensure that simulated conditions accurately represent real-world engine behaviour.
• Dataset helps validate the simulation model and increases confidence in the accuracy of the results obtained. Researchers can use this data to validate and benchmark combustion models, evaluate alternative fuel blends, optimize nozzle designs, and improve simulations. The specificity of the data set allows direct application to studies focused on heat transfer, engine performance, and optimization under similar operating conditions.

## 2 Background

Approximately one-third of the total energy input into the engine is lost to the environment through heat transfer. Temperatures in the combustion chamber of an engine can rise to around 2700 K, and the materials used [1,2] to build the engine cannot withstand such high temperatures for long periods of time. Proper heat transfer is essential to maintain the function and durability of the engine and to prevent degradation due to excessive heat.

Engine heat dissipation is critical to maintaining efficiency and longevity [[3], [4], [5]]. With a complex heat transfer system, a detailed study is required to better understand it [6,7]. The subsystems include the intake system, with variables such as the intake port, intake valve, and intake manifold, which are critical even in the intake phase. During compression, heat is transferred from the cylinder walls to the combustion gas [[8], [9], [10]]. At the point of combustion, the gas temperature rises significantly and expands, increasing its velocity and decreasing its temperature. This is when the rate of heat transfer is at its highest [3,11].

A study of the effects of injector hole number and injection pressure on diesel engines found that the number of injector holes affects fuel atomization, engine efficiency and emissions. Increasing the number of holes improves fuel properties and engine performance [12]. Another study [13], combining experimental and simulation approaches, showed that the number of injector holes affects combustion rate, NOx emissions, and soot emissions. Higher hole counts can result in increased soot formation. Overall, studies have shown that the number of injector holes affects fuel distribution, combustion efficiency, and performance, and studying it helps optimize engine performance and reduce emissions [14].

The novelty of this study lies in the use of dynamic grids in the chamber modeling section of the AVL-Fire software to simulate the intake and combustion processes, comparing pressure data with experimental results for different injector configurations. In addition, the effect of blending hydrogen with diesel fuel on the heat transfer coefficient is investigated, addressing the need for lower emissions and alternative fuel sources in diesel engines.

## 3 Data Description

This research investigates the effect of varying the number of injector holes on the heat transfer coefficient and pressure within the combustion chamber of a hydrogen-diesel engine. Using AVL FIRE software with a coupled Chemkin mechanism, simulations explore the effects of different fuel compositions (10 % hydrogen and 90 % diesel) and nozzle configurations (1, 3, and 6 holes) on engine performance. AVL FIRE software is a powerful computational fluid dynamics (CFD) tool specifically designed to simulate combustion processes in internal combustion engines. It allows detailed analysis of combustion phenomena, emissions and performance. Chemkin Mechanism is a software package that provides comprehensive chemical kinetic models for combustion simulation. It helps to accurately predict combustion behavior by taking into account detailed chemical reactions and species interactions within the combustion process. The integration of Chemkin mechanisms in AVL FIRE increases the accuracy and reliability of combustion simulations for engine design and optimization. The study focuses on a 4-cylinder single-hole injector diesel engine to analyze how the number of injector nozzles affects the cylinder pressure and convective heat transfer coefficient distribution. Dynamic mesh generation for intake and exhaust ports, along with the combustion chamber using AVL-Fire's chamber modeller section, facilitates the simulation of intake and combustion processes. Pressure data comparisons are made at 2800 rpm with different hole injectors, and the convective heat transfer coefficient distribution over the chamber walls is evaluated using a time step method, comparing the results with experimental data from Woschni and Hohenberg.

### 3.1 Data set mesh generation

**Fig. 1:**
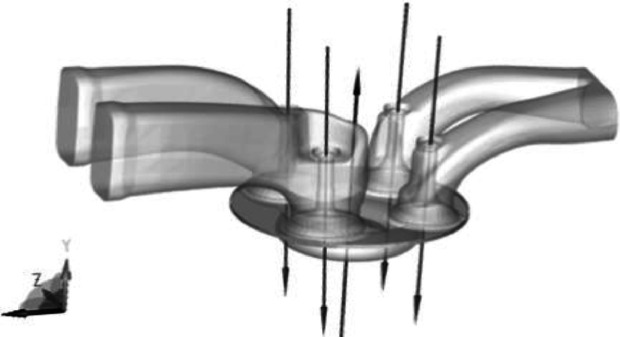
Combustion chamber geometry.

**Fig. 2:**
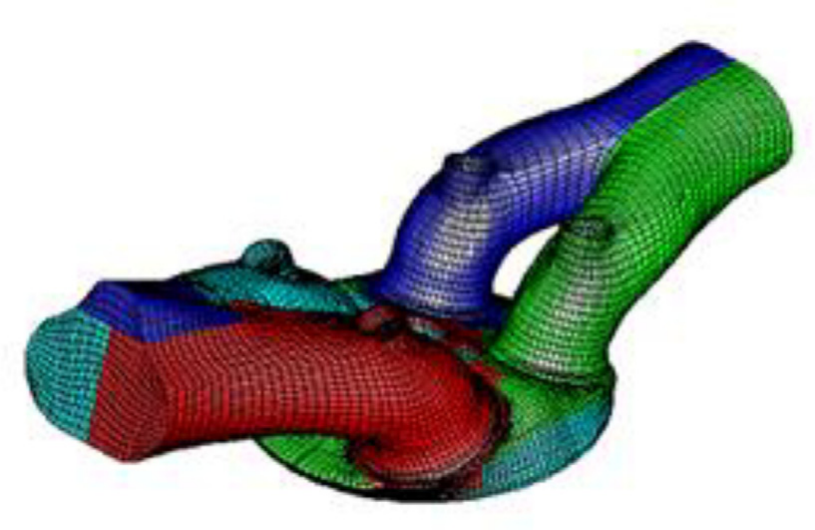
Creating the mesh for the overlap state of the valves for the chamber geometry.

Meshing is an essential part of the computational fluid dynamics (CFD) process for simulating heat transfer in combustion chambers. The geometry is first structured into different components, such as the bed, the valve seat surface, and the exhaust and intake valves. Meshes are generated using commercial software, such as AVL FIRE and Chamber modeler, and then combined in the model assembler. Fig. 1, Fig. 2 show the geometry of the combustion chamber and the mesh generation for the overlapped state of the valves. The total number of cells is approximately 1220,000.

**Fig. 3:**
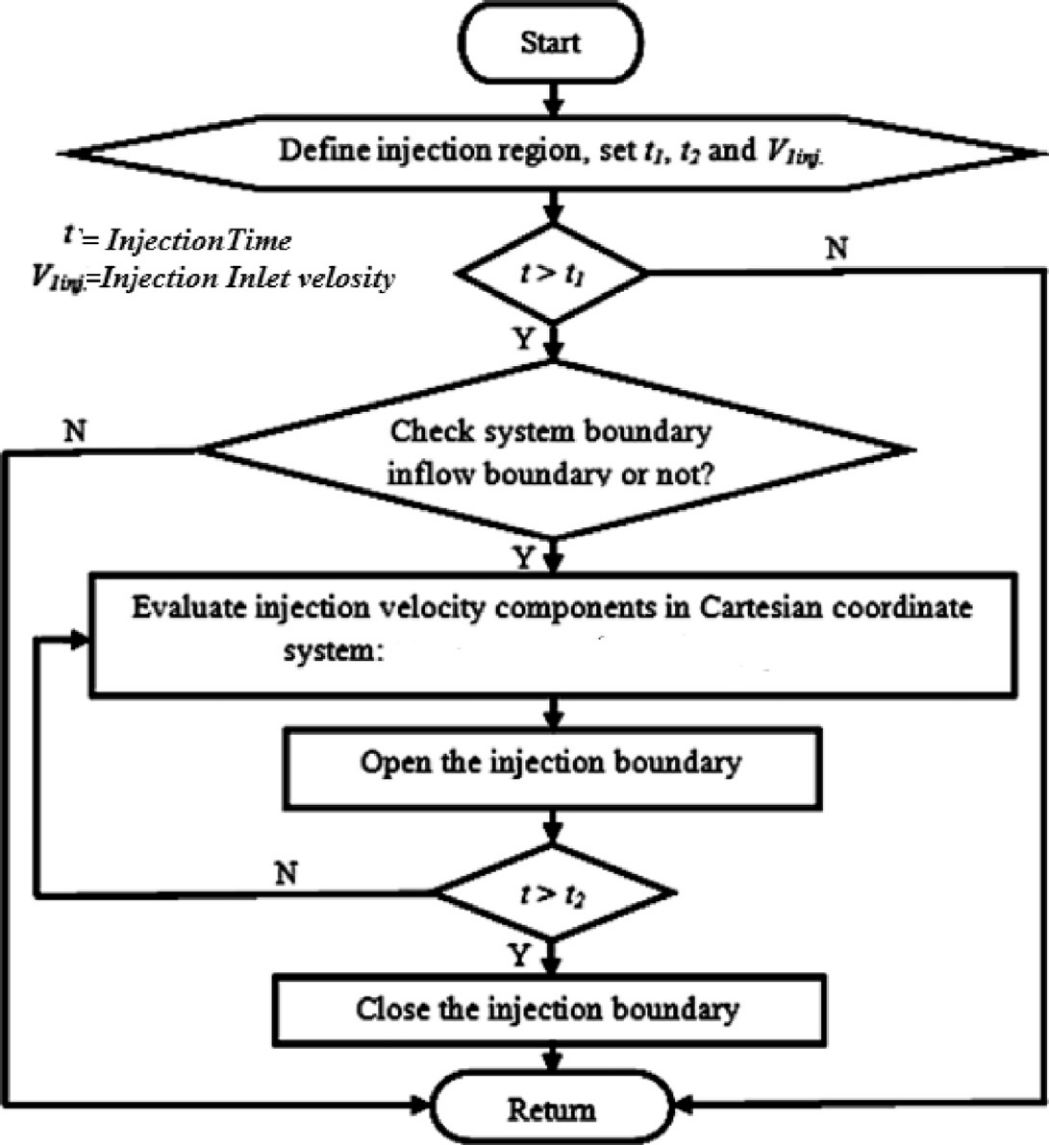
Flowchart for the injection program.

Fig. 3 shows Flowchart related to the fuel injection program in combustion chamber in a diesel engine. It was incorporated with the main AVL Fire code to complete the multidimensional model. Chemkin mechanism simulates the fuel injection process as a boundary condition. Chemkin sets the inlet velocity to value from experimental tests at the beginning of fuel injection through the velocity open boundary.

## 4 Experimental Design, Materials and Methods

General research on combustion chambers indicates that variations in temperature and velocity lead to uneven heat flux distribution on the chamber walls. Stable heat transfer within the cylinder combustion chamber is governed by specific equations. While radiation plays a minor role in gasoline engines, it is significant in diesel engines. Total heat transfer is sufficient for many applications, but understanding the instantaneous heat flux is critical for accurate simulations. This highlights the importance of considering factors such as turbulence intensity at air and fuel inlets, as they significantly affect temperature fields, radiative heat sources, and heat transfer rates at chamber walls. Incorporating these findings into combustor design and simulation can improve efficiency and performance while reducing emissions [15]. The total heat transfer is usually sufficient for certain applications, but the instantaneous heat flux is essential for effective simulation.

(1) $$q=\overset{˙}{{\dot{q}}_{cv}}+\overset{˙}{{\dot{q}}_{r}}=h_{c.g}\left(\bar{T_{g}}-T_{w.g}\right)+\sigma \varepsilon \left(\bar{T_{g}^{4}}-\bar{T_{w.g}^{4}}\right)$$

This equation correlates the heat flux ($\overset{˙}{q}=\frac{\overset{˙}{Q}}{A}$) with the measured temperature ($T_{w.g}$)

For the initial state, the temperature and pressure in the combustion chamber are assumed to be the same as the ambient temperature and pressure (T=300k,P=0.957bar). The piston is initially at the top dead center (TDC) point, and the flow turbulence pattern selected for the simulation is the k-epsilon model, which takes into account the rotational flow and the rapid movement of the piston. The initial kinetic energy in the chamber is set to$\;5\;\frac{m^{2}}{s^{2}}$ [16].

The first step in the mesh generation process for fluid dynamics simulations is to structure the geometry of the combustion chamber by dividing it into different components such as the bed, valve seat surface, exhaust valves, and intake valves. Meshes are then created for each component using specialized software such as AVL FIRE software and Chamber Modeler. These component meshes are then incorporated into the model assembler section to complete the mesh generation process. This approach ensures that the geometry is accurately represented in the simulation, allowing for detailed analysis of fluid-structure interactions. The use of dedicated software tools helps to efficiently generate meshes tailored to each component of the combustor, increasing the overall accuracy and effectiveness of the simulation.

**Fig. 4:**
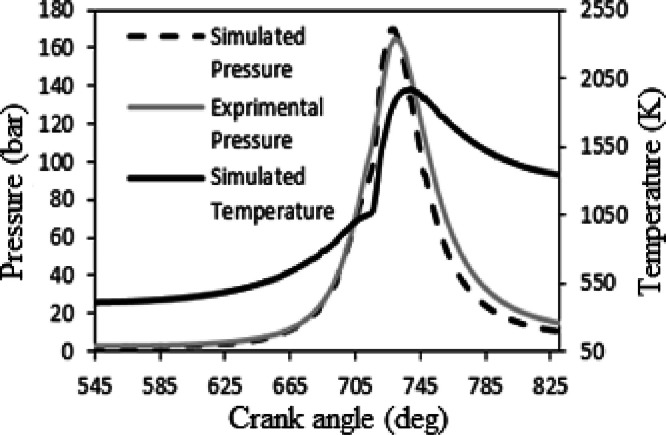
Analysis of the agreement between the temperature and pressure results obtained from experimental data and those obtained from numerical simulations.

The experimental validation process involves comparing pressure and temperature data inside the chamber with actual engine performance at 2800 rpm. The results show excellent pre-ignition agreement, with less than 1 % deviation from experimental values. Fig. 4 illustrates this comparison, showing pressure and temperature trends within the chamber. This meticulous validation process ensures the accuracy and reliability of the software-generated data by closely matching it to real engine operation. The validation process is critical to confirming the accuracy of simulation results against real-world engine performance metrics, thereby increasing the credibility of the software's predictive capabilities.

A temperature range of 670 to 850 °Celsius (50 °CA bTDC to 130 °CA aTDC) was selected for validation. Initially, at the start of fuel injection at 51 °CA bTDC to produce a premixed mixture, combustion is minimal, resulting in insignificant changes in the average heat transfer coefficient due to small temperature variations. Then, with the primary fuel injection at 18 °CA bTDC, combustion is initiated at the outer edge of the sprayed jet. This sequential process highlights the importance of temperature control and injection timing in achieving efficient combustion and heat transfer in CI engines, which is critical for model validation and performance optimization.

The geometric specifications and working conditions of the simulated and experimented engine are according to Table 1 (Table 2, Table 3, Table 4, Table 5, Table 6, Table 7, Table 8).

**Table 1:** Engine specification.

Engine	Perkins 1103A-33TG1		
Number of cylinders	4	Cycle	4 Stroke
Bore and Stroke (mm)	102 × 120	Length (cm)	124.25
Compression Ratio	17:1	Number of nozzle holes	1
Displacement (L)	3.922	Engine speed maximum	4000 RPM
Maximum Power	47 kW	Start of pre- injection	47 °CA BTDC
Aspiration	turbocharged	Start of main injection	18 °CA BTDC
Combustion system	direct injection

**Table 2:** The effect of nozzle hole number and diesel fuel on Heat transfer coefficient.

Crank angle	1-hole injector	3-hole injector	3-hole injector
330	1010	1012	1025
430	996	997	999
530	994.5	995	998
630	1154	1162	1167
695	8856	8761	8762
720	10,189	9845	9710
723	10,170	9890	9740
725	10,120	9832	9790
750	8140	8348	8040
790	1254	1254	1252
830	1111	1132	1134

**Table 3:** The effect of nozzle hole number and diesel fuel on in- cylinder pressure.

Crank angle	1-hole injector(diesel fuel)	3-hole injector(diesel fuel)	6-hole injector(diesel fuel)
650	40.37	40.34	40.33
675	53.4	53.4	53.4
700	69.3	69.3	69.3
724	183	176	167
750	78.32	72.76	71.873
775	38.2	38	37.95
800	37.2	37.1	37

**Table 4:** Effect of number of nozzles on fuel spray penetration depth versus crank angle.

Crank angle	3-hole injector(H2+diesel fuel)	3-hole injector(H2+diesel fuel)	6-hole injector(H2+diesel fuel)
650	40.37	40.34	40.33
675	53.4	53.4	53.4
700	69.3	69.3	69.3
724	194	183.5	174.2
750	78.32	72.76	71.873
775	38.2	38	37.95
800	37.2	37.1	37

**Table 5:** The effect of nozzle hole number and diesel fuel and hydrogen on in- cylinder pressure.

Crank angle	1-hole injector(H2+diesel fuel)	3-hole injector(H2+diesel fuel)	6-hole injector(H2+diesel fuel)
650	0	0	0
675	0	0	0
690	0.0045	0.0035	0.0025
700	0.0235	0.02	0.017
710	0.0267	0.026	0.0251
720	0.0266	0.0264	0.0264
725	0.0266	0.0264	0.0264
730	0.0266	0.0264	0.0264

**Table 6:** The effect of nozzle hole number and diesel fuel on in- cylinder temperature.

Crank angle	1 hole injector	3-hole injector	6-hole injector
545	510	523	531
585	632	645	652
625	784	796	823
665	875	905	932
705	934	956	962
725	905	932	943
745	1856	1890	1910
785	1432	1510	1519
825	1234	1323	1341

**Table 7:** The independence of the results from the number of grid cells.

Time	1 million cell	1.2 million cells	1.4 million cells
12	147.3	148.3	148.49
14	152.37	154.78	154.9
16	157.4	162.3	162.9
18	162.1	165.6	166.1
20	165.1	168.289	168.45
22	168.3	171.3	171.4

**Table 8:** The effect of the number of injector nozzles on the heat transfer coefficient (Maximum heat transfer coefficient (W/m^2^.k).

1-hole Nozzle(H2+diesel fuel)	3-hole Nozzle(H2+diesel fuel)	6-hole Nozzle(H2+diesel fuel)	1-hole Nozzle(diesel fuel)	3-hole Nozzle(diesel fuel)	6-hole Nozzle(diesel fuel)
10,189	9870	9805	10,028	9720	9710

## Limitations

Research limitations are primarily related to the fuel composition prior to injection, which requires specific technology and equipment. In addition, limitations arise from the design and positioning angle of the injector within the combustion chamber. In certain combustion chambers, injector placement and angle are critical factors that are directly influenced by chamber geometry. Therefore, by addressing these limitations during engine design, it's possible to mitigate potential problems and anticipate the development of an optimal engine with improved efficiency.

## Ethics Statement

Authors have read and agree to abide by the ethical requirements for publication in Data in Brief and confirm that the current work does not involve human subjects, animal testing, or data collected from social media platforms.

## CRediT authorship contribution statement

**Javad Zareei:** Writing – review & editing, Writing – original draft, Visualization, Validation, Supervision, Software, Resources, Project administration, Methodology, Investigation, Funding acquisition, Formal analysis, Data curation, Conceptualization. **Jose Ricardo Nuñez Alvarez:** Writing – review & editing, Writing – original draft, Software, Resources, Investigation, Formal analysis, Data curation, Conceptualization.

## Data Availability

Dataset of the effect of the number of injector holes on the heat transfer coefficient and the pressure in the combustion chamber of a hydrogen-diesel engine (Original data) (Mendeley Data)
